# Progress in Medicine

**Published:** 1898-09-24

**Authors:** 


### PROGRESS IN MEDICINE.

### DISEASES OF DIGESTIVE ORGANS.

(*Continued from page* 411.)

**Dilatation of the Stomach.**—At the Harveian Society, Armstrong[13] introduced a discussion on this disease. In treatment, he aims at keeping down fermentation, distending the stomach as little as possible, and increasing its motor power. He gives fuming hydrochloric acid 6-12 drops in six ounces of water as an antiseptic, nerve tonics, and Carlsbad salts as an evacuant. Local massage and electricity are disappointing, but galvanisation of the higher centres and the needle bath are sometimes useful. Very little fluid should be taken with meals, and farinaceous food and vegetables should be avoided. Hare states that emetics, such as pulvis ipecacuanha, are much more potent remedies than lavage of the stomach, as they cause forcible contractions of the stomach, and these squeeze out of the glands the morbid and retained secretion. Ewart recommends that patients suffering from moderately severe dilatation should be kept in bed, with the feet raised to prevent any dragging of the viscus, and that a belt should be worn afterwards as a support. Simple atony of the stomach must be distinguished from confirmed dilatation, and this again from that form where fibrous tissue has replaced the muscular. Armstrong has not found postural treatment of value, nor the belt of much use in true dilatation. Gastro-enterostomy is desirable in very bad cases. An instance of acute dilatation is reported by Box and C. S. Wallace,[14] as following a blow on the epigastrium. Peritonitis was suspected, and the abdomen was opened. The stomach was found distended, and filling the whole of the abdomen. The viscus was opened and emptied, but death followed in a few hours. No cause was found, post mortem, for the distension. Diverticula, or localised dilatations, are sometimes due to pressure of foreign bodies, sometimes from an injury, and sometimes from the results of an ulcer. Ferguson,[15] at Glasgow, showed instances of some of these deformities, and mentions also that caused by adhesions. One specimen, which he showed, could not be accounted for by any of these methods, and he regards it as a pulsion diverticulum, which is essentially a hernia of the mucous membrane through the muscular walls. The patient was a voracious eater, and drunkard, and the stomach, besides general dilatation, showed patches of atrophy of the muscular coat. One of these had probably given way, and produced this diverticulum near the cardiac orifice on the upper and outer aspect of the organ. Bettman[16] gives the results of an investigation upon the normal shape of the stomach. No organ, he says, varies so much in form as the stomach. In one person he finds a cylindrical type, resembling a long drawn-out tube; in another the vertical diameter is nearly the same as the long one. Nor is this the effect of long-continued use, for the same variations are found in fœtal stomachs. Thus the form depends on antenatal conditions, and differs for individuals just as their ears do. Contrary to the statements of the text-books, the fœtal stomach has often a fundus as well developed as in adult life, and the cylindrical shape is not more common than in adults. Indeed, the fundus is more developed in infants and young children than at any other period of life. He also finds from his examination of stomachs inflated with air that the œsophagus enters the stomach much nearer the anterior wall than the posterior. He considers that the active movements of the stomach are confined to the pyloric half, and that the hour-glass stomachs, with median constrictions, of which about 100 have been reported, merely accentuate the normal difference between the quiescent cardiac portion and the propulsive part near the pylorus.

**Spasmodic Stricture of the Cardiac End of the Stomach** is the subject of an interesting paper by Soltau Fenwick.[17] It may occur in neurotic and anæmic persons, especially after a fright, or from irritation of one of the abdominal viscera. Catarrhal and other affections of the œsophagus, slight injuries, habitual drunkenness, and even organic diseases of the stomach may be followed by it. There is dysphagia, which varies in severity from day to day; pain and soreness is felt in swallowing, and deters the patient from eating, while in some cases we find œsophageal vomiting unattended with nausea or straining; and gaseous distension of the stomach, with retained air, is noticed in others. Great emaciation and exhaustion from want of food is rarely seen. A diagnosis is usually easy, from the sudden commencement and varying character of the symptoms, the age of the patient, and the absence of a permanent obstruction to the passage of a bougie. Dysphagia and regurgitation of food may also arise in middle life from the presence of a diverticulum of the œsophagus. This is soon accompanied by a swelling in the neck where the pouch lies, and compression of it results in some of the food flowing back into the mouth. A tube, when inserted into the throat, will be arrested if it enters the pouch at a distance of six or seven inches from the incisor teeth. The treatment of spasmodic constriction depends on improving the general health, while many cases are cured by introducing a full-sized bougie daily, and leaving it *in situ* for five minutes. The instrument may be anointed with cocaine ointment if much pain is experienced.

**Gastroptosis.**—Langerhaus[18] employs for diagnosis a stomach tube with a soft rubber balloon at the end, which is distended when in the stomach with a measured amount of air. The anterior abdominal wall is then seen in gastroptosis to arch forward about the level of the umbilicus. He believes the affection is caused, not by tight lacing, but by parturition, anæmia, or nervous dyspepsia, and it interferes with the motor power of the stomach, besides causing pressure on and irritation of the neighbouring organs. In the form produced through parturition alone abdominal bandages are of service. Massage of the lumbar muscles or faradisation of the abdomen seem useless, but gymnastic exercises and nerve tonics, and occasionally fixation of the kidneys, appear to give good results. Bial,[19] besides recommending a suitable binder, lays stress on the treatment of the nervous system. The condition is common in women, and may practically exist without symptoms if the general health is good and a neurotic state be prevented.

[13] Lancet, Mar. 26. [14] Lancet, June 14. [15] Glasgow Med. Jour., Mar. [16] Amer. Jour. Med. Sci., June. [17] Brit. Med. Jour., April 30. [18] Med. Rec., April 23. [19] Brit. Med. Jour., April 2.

